# Ileocolic Enteroenteric Fistula in Small Bowel Obstruction With Cecum Perforation in the Pediatric Age Group: A Case Report of a Rare Complication

**DOI:** 10.7759/cureus.75995

**Published:** 2024-12-19

**Authors:** Survesh k Gupta, Umesh K Gupta, Rafey A Rahman, Anil Kumar

**Affiliations:** 1 Department of Pediatric Surgery, Uttar Pradesh University of Medical Sciences, Saifai, IND; 2 Department of General Surgery, Uttar Pradesh University of Medical Sciences, Saifai, IND

**Keywords:** cecum perforation, enteroenteric, ileocolic, intestinal fistula, obstruction

## Abstract

Enteroenteric fistula in the pediatric age group is an unusual presentation. It can create a diagnostic dilemma for the physician, particularly in the absence of any previous surgery, prolonged abdominal symptoms, or inflammatory bowel disease. The patient is a 10-year-old girl who presented with mild-grade fever, abdominal distension, scanty stool passage, and foul-smelling vomiting for the past 10 days. Before this, the patient had received treatment for 20 days for her fever and diarrhea from a quack, and her symptoms had improved. There was no history of prolonged fever, chronic diarrhea, weight loss, blood in the stool, trauma, foreign body ingestion, or hospitalization for any medical or surgical condition. After resuscitation, the patient was evaluated with blood tests, serum tests, and other necessary investigations, and surgery was planned. Intraoperative findings included pyoperitoneum with dilated small bowel. The omentum was densely adhered to the distal ileum, causing distal ileal obstruction, with a perforation approximately 1 cm in diameter in the cecum. The appendix appeared normal. Ten centimeters proximal to the ileocecal junction, there was a nondistensible and redundant segment of the distal ileum with a nondissectible gangrenous omentum. This segment was causing complete obstruction of the small bowel contents. Just proximal to this segment, an ileotransverse colon fistula was present. A double-barrel ileostomy was created for the patient, and she was discharged on postoperative day 10. In pediatric intestinal obstruction, common causes include simple band obstruction, Meckel’s band obstruction, intestinal adhesions, intussusception, and complicated appendicitis, among others. However, surgeons may encounter the unusual presentation of an enteroenteric fistula as a cause of intestinal obstruction.

## Introduction

A fistula is an abnormal passageway that forms between two organs in the body or between an organ and the exterior of the body [1]. As the definition suggests, a fistula connects two different surfaces or lumens. Common types of intestinal fistulas include enterocutaneous, enteroenteric, enterovesical, enterocolic, enteroatmospheric, and rectovaginal fistulas [2]. Enteric fistulas are a complex and challenging complication following bowel surgery, often associated with high morbidity and mortality, prolonged hospital stays, and increased healthcare costs [2]. Enteroenteric fistulas are abnormal connections between different segments of the intestine, and their management can be particularly challenging due to their complex anatomical locations and associated physiological effects. The presentation of perforation peritonitis can vary significantly. In most cases, it is an acute condition requiring emergency laparotomy. However, in rare instances, factors such as the site of perforation, immune response, and omental reaction can result in a delayed and more complicated presentation [3]. This case report highlights a unique instance of a distal ileotransverse colonic fistula in a patient with small bowel obstruction caused by a concealed cecal perforation.

## Case presentation

A 10-year-old female child presented to the pediatric surgery emergency department with chief complaints of mild-grade fever, abdominal distension, scanty stool passage, and foul-smelling vomiting for the past 10 days. The patient had been asymptomatic one month prior when she developed mild-grade fever, diarrhea, and nonbilious vomiting. She received medical treatment for her fever and diarrhea from a quack for 20 days, after which her symptoms were relieved. However, the patient subsequently developed abdominal distension and bilious vomiting, leading to her admission and medical management elsewhere for 10 days. During this period, she passed stool only three to four times and experienced an episode of occult blood in her stool. Despite this, her abdominal distension progressively worsened, accompanied by foul-smelling vomiting. There was no history of prolonged fever, chronic diarrhea, weight loss, blood in the stool, trauma, foreign body ingestion, or hospitalization for any medical or surgical condition before one month ago. There was also no history suggestive of tuberculosis or contact with a person who has tuberculosis. The patient had a history of pica and passage of worms with stool.

**Figure 1:**
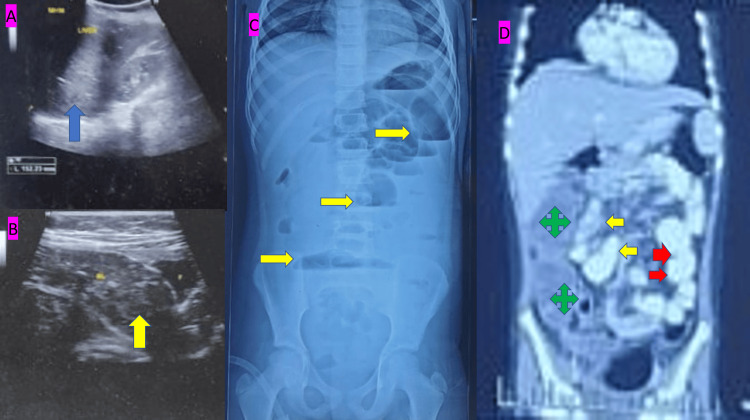
Preoperative USG, X-ray, and CECT of the abdomen of the patient. (A) USG of the abdomen showing enlarged liver (blue color filled arrow). (B) USG of the abdomen showing dilated bowel loop with fecalith in situ (yellow color filled arrow). (C) X-ray of the erect view of the abdomen showing multiple air-fluid levels (yellow color filled arrow). (D) CECT showing contrast in the small bowel (yellow color filled arrow), a contrast in the left side of the large bowel (red color filled arrow), and the right side of the large bowel showing nonfilling of contrast (green color filled plus sign) USG: ultrasonogram; CECT: contrast-enhanced computed tomography

On examination, the patient was febrile and dehydrated, with poor general condition. Pallor and abdominal distension with tenderness were noted. The nasogastric aspirate was found to be fecal. The patient was resuscitated with IV fluids and IV antibiotics. Blood investigations revealed severe anemia (4.4 g/dL) and dyselectrolytemia along with a deranged coagulation profile for which packed red blood cells, FFP, and other supportive treatment were given. Preoperatively, ultrasound findings revealed mild hepatomegaly and fecal-loaded, distended bowel loops with reduced peristalsis, suggestive of subacute intestinal obstruction (Figures 1A, 1B). An X-ray of the abdomen showed dilated bowel loops with multiple air-fluid levels and a paucity of bowel gas in the pelvis (Figure 1C). A contrast-enhanced computed tomography scan of the abdomen was performed, which indicated small bowel obstruction with mild enhancing wall thickening involving the terminal ileum, ileocecal junction (ICJ), and cecum. Multiple small to mildly enlarged mesenteric lymph nodes were scattered throughout the abdomen, predominantly in the right iliac fossa region, with mild ascites (Figure 1D).

**Figure 2:**
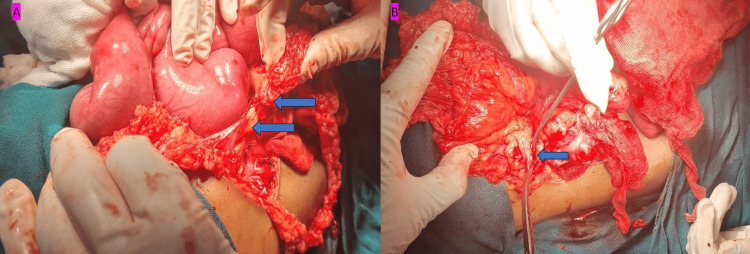
Intraoperative image of the patient. (A) Small bowel was densely conglomerated in the right iliac fossa (blue color filled arrow). (B) Perforation in the cecum (blue color filled arrow)

After resuscitation and stabilization of the patient, laparotomy was performed through a right transverse supraumbilical incision. Intraoperative findings included pyoperitoneum with dilated jejunum and proximal ileum; the omentum was densely adhered to the distal ileum, causing distal ileal obstruction. The small bowel was densely conglomerated in the right iliac fossa (Figure 2A). The bowel was delineated with the help of manual and hydrodissection. After dissection, a perforation approximately 1 cm in diameter was visualized in the cecum, with gangrenous margins (Figure 2B).

**Figure 3:**
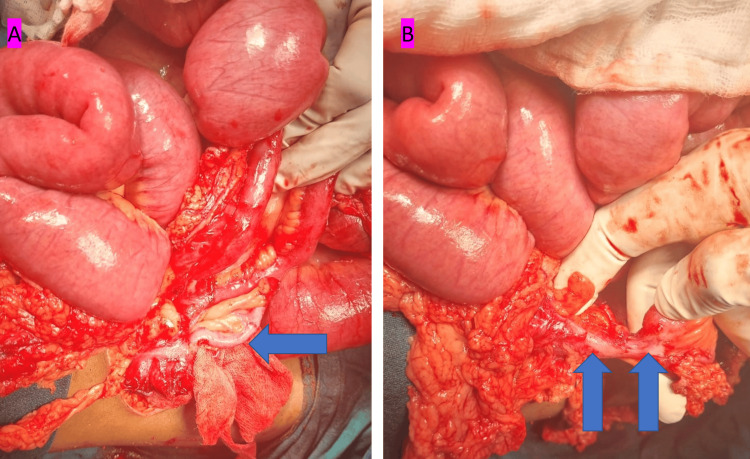
Intraoperative image of the patient. (A) Normal appendix (blue color filled arrow). (B) Ileotransverse colon fistula (blue color filled arrow)

The appendix was normal (Figure 3A). Ten centimeters proximal to the ICJ, approximately 10 cm of the distal ileum was nondistensible and redundant, with nondissectible gangrenous omentum. This segment was causing complete obstruction to the flow of small bowel contents. Just proximal to this segment, an ileotransverse colon fistula was present (Figure 3B).

Disconnection of the ileocolic fistula, along with resection of the 10 cm redundant ileal segment, was performed. After freshening the margins, the cecal perforation and the site of the transverse colon fistula were repaired. Since the repair of the cecal perforation was adjacent to the appendix, an appendectomy was also performed. Peritoneal lavage was done, and a double-barrel ileostomy was created approximately 10 cm proximal to the ICJ. Postoperative blood investigations revealed features of disseminated intravascular coagulation (DIC; international normalized ratio 1.08, PT 15 seconds, activated partial thromboplastin time 27.2 seconds, fibrinogen 614 mg/dL, D-dimer 1.68 µg/mL) (Table 1). The echocardiogram was normal, but the electrocardiogram showed sinus tachycardia and nonspecific ST changes. The patient was in the DIC; therefore, Ecosprin and cilostazol were started.

**Table 1:** Postoperative coagulation profile of patients INR: international normalized ratio; PT: prothrombin time; APTT: activated partial thromboplastin time

Coagulation profile component	Observed value	Reference value
INR	1.08	1-1.5
PT	15 second	11.7-15.3 second
APTT	27.2 second	24-32 second
Serum fibrinogen	614 mg/dL	200-400 mg/dL
D-dimer	1.68 µg/mL	0-0.5 µg/mL

Postoperatively, on day 2, the stoma was functional, and the patient was allowed oral intake on postoperative day 3. A surgical site infection was present, which was managed conservatively with saline irrigation and dressing. The patient was discharged on the 10th postoperative day. The histopathological examination (HPE) of the sent specimen revealed gangrenous ileum with ulcerated mucosa, marked hemorrhagic exudate, necrosis, and epithelial cell granulomas. The HPE of the mesenteric lymph node showed Langerhans-type giant cells, which were negative for acid-fast bacilli. The appendix appeared unremarkable on HPE.

## Discussion

To the best of our knowledge and based on our literature search, this is a rare case report of an ileotransverse colon enteroenteric fistula in a pediatric patient with no prior typical history of inflammatory bowel disease (IBD). In adults, enteroenteric fistulas have been reported in cases of IBD and Koch’s cocoon abdomen, while in older age groups, these fistulas are often associated with malignancy [4,5]. Additionally, enteroenteric fistulas have been documented following the retention of intra-abdominal surgical sponges [6-8]. Although a few cases of enteroenteric fistulas in pediatric patients have been reported, they are typically linked to foreign body ingestion [9-11]. In neonates, these fistulas are frequently associated with postnecrotizing enterocolitis or meconium aspiration [12]. In our case, the likely cause of the enteroenteric fistula could be either IBD or a possible missed cecal perforation. The exact event leading to the formation of this fistula is controversial due to the delay in reaching a tertiary healthcare center. However, the HPE supports the diagnosis of IBD. In the past two decades, there has been a notable rise in the incidence of IBD in South and Southeast Asia. Although the prevalence of IBD in India is lower compared to Western countries, given its population of 1.3 billion, the disease burden appears to be among the highest globally [13]. Morphological factors associated with IBD may include more severe involvement in the proximal regions, involvement of the terminal ileum, active appendicitis, and a prominent presence of neutrophils in the lamina propria [14]. Foreign body ingestion typically presents with gastrointestinal symptoms and tenderness in the lower abdomen. Delays in diagnosis and management can lead to worse outcomes in pediatric patients, as children may struggle to communicate their discomfort unless symptoms are clearly apparent [9]. In our case, there was a delay in diagnosis because the child had previously been treated conservatively for obstruction elsewhere. One study found that enteroenteric fistulas were associated with intestinal perforation in 50% of cases [15]. Aside from foreign body ingestion, a history of surgery, or tuberculosis, intestinal fistulas in the pediatric population are quite rare. An enteroenteric fistula following obstruction is also uncommon. For example, an 89-year-old female patient with a history of excessive analgesic use was reported to have small bowel obstruction secondary to an enteroenteric fistula [16].

## Conclusions

There are a few case reports of pediatric patients with IBD presenting with enteroenteric fistulas. In such cases, symptoms like bilious vomiting and, in later stages, fecal vomiting indicate the need for emergency surgical intervention. In this case, the brief clinical history of intestinal obstruction, along with intraoperative findings of an ileocolic enteroenteric fistula, underscores the need for a thorough assessment of any previous acute abdominal episodes. It is crucial to evaluate whether there is any history indicative of IBD or tuberculosis, especially in the absence of acute abdominal symptoms.
